# Prediction of tissue-specific cis-regulatory modules using Bayesian networks and regression trees

**DOI:** 10.1186/1471-2105-8-S10-S2

**Published:** 2007-12-21

**Authors:** Xiaoyu Chen, Mathieu Blanchette

**Affiliations:** 1 McGill Centre for Bioinformatics. 3775 University Street, room 332, Montreal, Quebec, Canada, H3A 2B4; 2 Department of Computer Science and Engineering, University of Washington, Seattle, WA 98105, USA

## Abstract

**Background:**

In vertebrates, a large part of gene transcriptional regulation is operated by cis-regulatory modules. These modules are believed to be regulating much of the tissue-specificity of gene expression.

**Results:**

We develop a Bayesian network approach for identifying cis-regulatory modules likely to regulate tissue-specific expression. The network integrates predicted transcription factor binding site information, transcription factor expression data, and target gene expression data. At its core is a regression tree modeling the effect of combinations of transcription factors bound to a module. A new unsupervised EM-like algorithm is developed to learn the parameters of the network, including the regression tree structure.

**Conclusion:**

Our approach is shown to accurately identify known human liver and erythroid-specific modules. When applied to the prediction of tissue-specific modules in 10 different tissues, the network predicts a number of important transcription factor combinations whose concerted binding is associated to specific expression.

## Background

A cis-regulatory module (CRMs) is a DNA region of a few hundred base pairs consisting of a cluster of transcription factor (TF) binding sites [1]. By binding CRMs, transcription factors either enhance or repress the transcription of one or more nearby genes. Coordinated binding of several transcription factors to the same CRM is often required for transcriptional activation, thus allowing a very specific regulatory control.

High-throughput experimental identification of CRMs remains inaccessible, especially for distal enhancers. Methods like genomic localization assays (also known as ChIP-chip) using whole genome tilling arrays may soon improve the situation, but the cost of such extremely large arrays will limit their utilization. Because of this, several computational approaches have been developed for predicting cis-regulatory modules. Some attempt to identify regulatory modules with a particular function (e.g. muscle [2] or liver [3] specific CRMs, and many others [4-6]) by building or learning a model of the binding site content of such modules, based on a set of known modules. These methods generally obtain a reasonable specificity, but their applicability is limited to the few tissues, cell types, or conditions for which sufficiently many experimentally verified modules can be used for training. Others seek more generic signatures of cis-regulatory regions, like inter-species sequence conservation [7], sequence composition [8], or homotypic and heterotypic binding site clustering [9,10]. These methods are more widely applicable, but their predictions may be of lesser accuracy, because they do not rely on any prior knowledge. Furthermore, the predictions made by these algorithms are not accompanied by any annotation regarding the putative function of the modules. The PReMod database [11] contains more than 100,000 human CRM computational predictions, mostly consisting of putative distal enhancers.

By adjoining other types of information to the predicted module information, additional insights can be gained into the function of specific modules. For example, in yeast, Beer and Tavazoie have used gene expression data to train a algorithm to predict expression data based on sequence information. In human, Blanchette et al. [12] and Pennacchio et al. [13] have used tissue-specific gene expression data from the GNF Altas2 [14] to identify certain transcription factors involved in tissue-specific regulation and Pennachhio et al. [13] have further developed models to predict the tissues-specificity of regulatory modules based on their binding site content. In this paper, we propose a new approach to the detection of tissue-specific cis-regulatory modules. Our algorithm uses a Bayesian network to combine binding site predictions and tissue-specific expression data for both transcription factors and target genes. It identifies the transcription factors and combinations thereof whose presence bound to a module appears to be resulting in tissue-specific expression. Our approach takes advantage of the facts that tissue-specific CRMs are likely 1) to be located next to genes expressed in that same tissue, 2) to contain many binding sites for TFs that are also expressed in that tissue, and (3) to contain binding sites whose presence in other modules also appears to be associated to tissue-specific expression. Our approach falls under the category of unsupervised learning, as it does not rely on any labeled training set or any type of prior knowledge regarding the TFs that may be important for a given tissue.

Importantly, the Bayesian network contains at its core a regression tree to represent the dependence between the regulatory activity of a CRM and the set of TFs predicted to bind it. A new unsupervised Expectation-Maximization-like algorithm is developed to infer the parameters of the network, including the structure of the regression tree. Our approach is related to that of Segal et al. [15,16] but differs in that it takes advantage of available TF position weight matrices and TF expression data to allow tissue-specificity predictions. Moreover, based on the candidate modules predicted by PReMod, our approach is allowed to detect distal enhancers that are involved in tissue-specific expression.

We show that our method is able to accurately discriminate between known liver and erythroid-specific modules, even in the presence of a large fraction of modules with neither function, by discovering important combinations of transcription factors associated to these tissues. When applied to a larger set of putative modules and tissues, several known tissue-specific TFs were recovered, and many interesting new TF combinations were predicted to be linked to tissue-specific expression.

## Methods

The goal of the method developed in this paper is to predict whether a given putative cis-regulatory module is responsible (at least in part) for the expression of a given gene in a particular tissue. Since the problem of predicting regulatory modules has already been studied extensively, we assume that a set of candidate CRMs $ℳ=\{M^{1},...,M^{\left|ℳ\right|}\}$ has been identified in the genome under consideration and we focus on determining their tissue-specificity. We emphasize that many of these predicted CRMs are likely to be false-positives (i.e. they have no regulatory function whatsoever), and most are probably not specific to any tissue; our goal is to identify those that are. Given a putative CRM *M*^*m*^, a gene *G*, and a tissue (or cell type) *T*, we want to determine whether module *M*^*m *^up-regulates gene *G *in tissue *T*. (We focus only on the identification of enhancers, rather than repressors, because it is difficult to distinguish between repressed genes and genes that are not expressed due to the lack of activators.) To this end, we define a Bayesian network that is used to combine various types of evidence, including the putative transcription factor binding sites contained in *M*^*m*^, the expression levels of the set of transcription factors predicted to bind *M*^*m*^, and the expression level of gene *G*.

Importantly, and perhaps counter-intuitively, we train a *single *Bayesian network that will be applicable to predicting tissue-specific regulatory modules in *all *the tissues considered. This stems from the hypothesis that the enhancer activity of a module should depend only on its binding site content and on the expression levels of the transcription factors binding it, and not directly on the tissue considered. By allowing sharing regulatory mechanisms across tissues, we hope to improve our sensitivity to subtle regulatory mechanisms. One obvious drawback of this method is that unobserved entities like the presence or absence of tissue-specific transcriptional co-activators may affect the regulatory effect of a given module in different tissues even if the set of TFs bound to it does not change.

**Figure 1:**
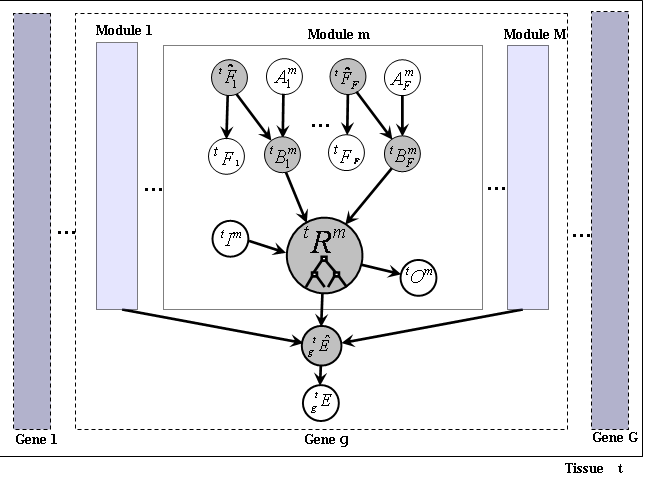
The bayesian network used for predicting tissue-specific regulatory modules. See section 'Bayesian network variables' for a description of the variables, and section 'Bayesian network architecture' for a description of their dependencies.

Typically, a Bayesian network consists of a set of observed variables, a set of unobserved variables, and an acyclic directed graph describing the direct dependencies between these. In this section, we first introduce the set of variables present in our network, which is depicted in Figure 1. We then describe the dependencies between these variables and the algorithms used to learn the parameters of the network.

### Bayesian network variables

Let Φ = {Φ_1_,...,Φ_|Φ|_} be a set transcription factors, let T={T1,...,T|T|} be a set of tissue (or cell) types, let G={G1,...,G|G|} be the set of all known human protein-coding genes, and let $ℳ=\{M^{1},...,M^{\left|ℳ\right|}\}$ be a set of predicted cis-regulatory modules. Since the notation describing the network requires many types of subscripts, we adopt the following convention: Right-subscripts refer to transcription factor indices; Right-superscripts refer to module indices; Left-superscripts refer to tissue indices; Left-subscripts refer to gene indices (for example, $\begin{array}{c} tissue\\ gene \end{array}X_{factor}^{module}$). We start by defining the observed variables for our network, shown in unshaded ovals in Figure 1. More detailed definitions pertaining to the specific data set analyzed in this paper will be found in Section 'Data sets'. Consider the following domains of index variables: 1 ≤ *m *≤ |ℳ|, 1 ≤ *f *≤ |Φ|, 1 ≤ *g *≤ |G|, and 1 ≤ *t *≤ |T|.

• $A_{f}^{m}$ is the real-number predicted affinity of transcription factor Φ_*f *_for module *M*^*m*^. It should reflect our confidence that, provided factor Φ_*f *_is expressed, it will bind module *M*^*m*^. It is a function of the number and the quality of Φ_*f*_'s predicted binding sites in *M*^*m*^.

• ^*t*^*F*_*f *_is a boolean variable describing whether transcription factor Φ_*f *_is expressed in tissue ^*t*^*T*.

• Egt is a boolean variable describing whether gene *g *is expressed in tissue ^*t*^*T*.

To model the relationships between the observed variables, it is necessary to introduce a set of hidden variables.

• $\begin{array}{c} t \end{array}{\hat{F}}_{f}$ is the actual state (active or inactive) of transcription factor Φ_*f *_in tissue ^*t*^*T*. State $\begin{array}{c} t \end{array}{\hat{F}}_{f}$ may not equal the observed expression level ^*t*^*F*_*f *_because of post-transcriptional regulation (e.g. activation due to external stimuli for nuclear receptors) or errors in the measurements of mRNA abundance.

• E^gt is the actual transcriptional status (transcribed or not transcribed) of gene _*g*_*G *in tissue ^*t*^*T*, which could be different from the observed mRNA abundance Egt because of mRNA degradation or errors in the measurements of mRNA abundance.

• $\begin{array}{c} t \end{array}B_{f}^{m}$ is a boolean variable indicating whether, in tissue ^*t*^*T*, module *M*^*m *^is bound by sufficiently many copies of factor Φ_*f *_for this factor to achieve its function.

• The fact that a module is bound by a transcription factor does not necessarily translate into this module being regulatorily active. Indeed, the presence of other transcription factors may be required for the module to become active. We represent the regulatory activity of module *M*^*m *^in tissue ^*t*^*T *by a boolean variable ^*t*^*R*^*m*^, which takes the value 1 when the module *M*^*m *^actively (and positively) regulates its gene. This is the variable whose value is of the most interest for predicting tissue-specific regulatory modules.

We acknowledge that using binary variables to represent expression levels and regulatory activity is a very crude approximation. Although all these variable should in theory be continuous, the quantitative relations between transcription factor expression levels, their binding affinity to a module, and the contribution of that module to the expression of the target gene remain poorly understood, so a more qualitative approach is preferable. Furthermore, due to the computational complexity of network inference, such a simplification was necessary. In fact, by reducing the size of the parameter search space, this simplification might actually be improving generalization from small data sets.

### Bayesian network architecture

In a Bayesian network, dependencies between variables are modeled as directed edges connecting the cause to the effect. The conditional probability of a node given the value of its parent(s) is described by a set of parameters that are either fixed or learned from the data. When the variables at hand have a finite domain, these conditional probabilities can be represented by a conditional probability table (CPT).

#### Conditional distributions of *E *and *F*

The observed expression levels *E *and *F *depend on the true expression levels $\hat{E}$ and $\hat{F}$ respectively. Since all variables are boolean, the conditional probability tables are the following:

$$\begin{array}{c} \mathrm{Pr}[E|\hat{E}]=\begin{array}{ccc} & E=0 & E=1\\ \hat{E}=0 & 1-\alpha_{E} & \alpha_{E}\\ \hat{E}=1 & \beta_{E} & 1-\beta_{E} \end{array}\\ \mathrm{Pr}[F|\hat{F}]=\begin{array}{ccc} & F=0 & F=1\\ \hat{F}=0 & 1-\alpha_{F} & \alpha_{F}\\ \hat{F}=1 & \beta_{F} & 1-\beta_{F} \end{array} \end{array}$$

Here, *α*_*E *_and *β*_*E *_are the probabilities of false-positive and false-negative in the discretized gene expression data, respectively. We assume that these parameters are shared among all genes, i.e. expression measurement errors are equally likely for all genes. Similarly, *α*_*F *_and *β*_*F *_are the probabilities that the discretized expression measurement for a given factor does not reflect their actual regulatory potency. Again, these parameters are shared among all transcription factors, although this might to be inaccurate for factors like nuclear receptors, which require external signals for activation.

#### Conditional distribution of *B*

The probability of $\begin{array}{c} t \end{array}B_{f}^{m}$, the random variable that describes whether module *M*^*m *^is bound by factor Φ_*f *_in tissue ^*t*^*T*, depends on whether the factor is expressed in that tissue, and on the affinity $A_{f}^{m}$ of the factor for that module. We assume that the parameters describing this conditional probability are the same for all *m *and *t*, so we drop some subscripts and superscripts to write Pr[*B*_*f*_|*A*_*f*_, *F*_*f*_]. We model this conditional probability indirectly, by instead modeling Pr[*A*_*f*_|*B*_*f *_= 1], the distribution of binding site affinities for a module that is bound, using a normal distribution with parameters *μ*_*f *_and $\sigma_{f}^{2}$ that will be estimated during training. Since the mathematical derivation is tedious (but relatively simple), it is left in Appendix 1.

#### Conditional distribution of *R *using regression trees

The most challenging set of conditional probabilities to represent is that of ^*t*^*R*^*m*^, which depends on the values of $\begin{array}{c} t \end{array}B_{1}^{m},...,\begin{array}{c} t \end{array}B_{\left|ℱ\right|}^{m}$. Again, we assume the parameters that describe this dependency are the same for all tissues ^*t*^*T *and all module *M*^*m*^, so we drop these indices. This assumption is equivalent to saying that the regulatory effect of the binding of a certain set of transcription factors does not depend on the module bound, the gene being regulated, or the tissue type.

**Figure 2:**
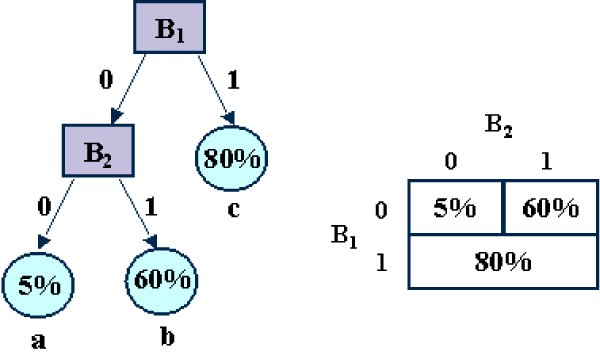
Example of a regression tree representing a small 2-variable conditional probability table.

How should we represent the probability that a module is regulatorily active, given the set of transcription factors bound to it, i.e. Pr[*R*|*B*_1_,...,$B_{\left|ℱ\right|}$]? Given that all variables are boolean, this conditional probability can be represented by a $2^{\left|ℱ\right|}$ × 2 CPT containing $2^{\left|ℱ\right|}$ parameters. In our application where ℱ contains several hundred transcription factors, this is obviously not practical, because (1) the CPT would be too large to store, and (2) we would need a huge amount of training data to learn the parameters. We thus use a more compact representation for this CPT, based on regression trees [17]. A regression tree is a rooted tree whose internal nodes are labeled with tests on the value of some variable *B*_*f*_. See Figure 2 for a small example. For boolean variables (our case here), each node *N *tests whether the some variable $B_{i_{N}}$ takes the value true or false. Each leaf *l *of the tree is associated with a probability distribution Pr[*R*|*l*]. Let $\pi (l)=\{B_{i_{1}}=b_{i_{1}},B_{i_{2}}=b_{i_{2}},...\}$ be the set of variable assignments obtained by following the path from the root to *l*. Let *l*(*b*_1_,...,$b_{\left|ℱ\right|}$) be the leaf reached when *B*_1 _= *b*_1_,...,$B_{\left|ℱ\right|}$ = $b_{\left|ℱ\right|}$. Then, the regression tree defines a complete conditional probability distribution: Pr[*R*|*B*_1 _= *b*_1_,...$B_{\left|ℱ\right|}$ = $b_{\left|ℱ\right|}$] = *p*(*l*(*b*_1_,...,$b_{\left|ℱ\right|}$)). When many of the *B*_*i*_'s are irrelevant to *R*, the representation is much more compact than the standard CPT and can be estimated from less data. We will jointly refer to the tree topology, the node labelings, and the probability distributions at the leaves as the meta-parameter Ψ. Inferring Ψ will be the most significant difficulty of this approach.

#### Conditional distribution of $\hat{E}$

The last set of dependencies is that of a gene's transcriptional activity E^gt on the regulatory activity of the neighboring regulatory modules. This raises the difficult question of determining which gene is being regulated by each module. This is relatively straight-forward when the module is located in the promoter region of a gene, but much less so when it is located 100 kb away from any gene. Here, for lack of more accurate information, we assume that a module *M*^*m *^only has the potential of regulating the gene _*g*_*G *whose transcription start site is the closest to it, denoted *closest*(*M*^*m*^). Then the expression level of gene _*g*_*G *depends on *regulators*(_*g*_*G*) = {*m*|*closest*(*M*^*m*^) = _*g*_*G*} = {*r*_1_, *r*_2_,...}. We will assume that the expression level of _*g*_*G *only depends on the number of its modules that are active, through a sigmoid function:

Pr⁡[E^g|Rr1,Rr2,...]=1/(1+e−b⋅(∑r∈regulators(Gg)Rr−a)), where *a *and *b *are user-defined parameters (see Appendix 3).

## Learning the network's parameters

Our Bayesian network contains a number of parameters whose values are not known a priori. We collectively refer to these parameters as $\Theta =\{\mu_{1},...,\mu_{\left|ℱ\right|},\sigma_{1}^{2},...,\sigma_{\left|ℱ\right|}^{2},\Psi \}$. The network will be trained using the set of all pairs (module, tissue). Let **A**, **E**, and **F **be the set of all TF affinity data, all gene expression data, and all TF expression data, respectively, over all tissues considered. A typical approach to estimating the network's parameters is to seek the value Θ* that maximizes the joint likelihood of the observed variables, i.e. Θ* = *argmax*_Θ_Pr[**A**, **E**, **F**].

An Expectation-Maximization algorithm can be used to learn the parameters Θ of the Bayesian network [18], whereby a local maximum of the likelihood function is reached by alternatively estimating the expected value of hidden variables given the observed variables and the current estimate Θ^0^, and then reestimating the maximum likelihood values for the parameters Θ. However, since Θ contains the tree structure, we cannot apply the standard EM algorithm for learning Bayesian networks, as this algorithm relies on the ability to analytically derive a maximum likelihood estimate for the parameters (see however [18]). Instead, a new EM-like algorithm with regression tree learning is developed to infer the tree within the network.

### Estimating posterior probabilities for hidden variables

Our first step is to calculate the expectation (or equivalently, the probability of taking the value 1, since all hidden variables are binary), for all hidden variables, given the value of the observed variables. These posterior probabilities can be calculated using the formulas given in Appendix 2. The derivation of most of these formulas is fairly straight-forward, except for the calculations involving the regression tree. Computing $\mathrm{Pr}[R|A,E,F]=\sum_{b\in {\left\{0,1\right\}}^{\left|ℱ\right|}}\mathrm{Pr}[R=r,B=b|A,E,F]$ can be done efficiently thanks to the regression tree representation.

### Maximum likelihood parameter estimation

Once the posterior probabilities of the hidden variables are computed, maximum likelihood estimators for the parameters of the network can be derived as given in Appendix 3. Again, the regression tree representing the dependence of *R *on *B*_1_,...,$B_{\left|ℱ\right|}$ poses significant challenge, as no efficient algorithm exists to choose the tree topology T. Instead, we developed a new tree learning algorithm, which adapts ideas from standard decision tree algorithms (e.g. C 4.5 [19], J48 [20]). The problem at hand is novel and challenging for several reasons:

1. Soft attributes: The input variables $\begin{array}{c} t \end{array}B_{f}^{m}$ are binary variables, but their values remain unknown at any given iteration of the EM-like algorithm. Only their distribution Pr[$\begin{array}{c} t \end{array}B_{f}^{m}$|**A**, **E**, **F**] is known for each *m*, *f *and *t*, given the current estimate of the parameters Θ.

2. Soft labels: The values of the target variables ^*t*^*R*^*m *^are also unknown, but their distribution Pr[^*t*^*R*^*m*^|**A**, **E**, **F**] is known.

#### Learning regression trees from probabilistic instances

Most decision tree learning algorithms are based on a greedy tree-growing approach trying to find the tree that minimizes the number of misclassifications [21]. Our tree learning algorithm is an adaptation of the standard approach using information gain as a method to select which attribute to select to split a node. Consider a node *N *that is currently a leaf and that we are considering splitting based on some attribute *B*_*i*_. The *weight *of a probabilistic instance $x=(\begin{array}{c} t \end{array}B_{1}^{m},...,\begin{array}{c} t \end{array}B_{\left|\Phi \right|}^{m})$ is the probability of the path from the root to *N*, under the attribute probability distributions given by *x*.

More precisely,

$$weight_{N}(m,t)=\prod_{\text{assignment }\Lambda \text{ on the path from root to }N}\mathrm{Pr}[\Lambda |A,E,F]$$

We can now define the weighted entropy at node *N *as:

$$weightedEntropy(N)=-\sum_{r=0,1}p_{r}\log_{2}p_{r},$$

where $p_{r}=\frac{\sum_{t=1}^{\left|T\right|}\sum_{m=1}^{\left|ℳ\right|}weight_{N}(m,t)\cdot \mathrm{Pr}[\begin{array}{c} t \end{array}R^{m}=r|A,E,F]}{\sum_{t=1}^{\left|T\right|}\sum_{m=1}^{\left|ℳ\right|}weight_{N}(m,t)}$, and *totalWeight*(*N*) = ∑_*t *_∑_*m*_*weight*_*N*_(*m*, *t*). Then, the information gain obtained by splitting a leaf *N *with attribute *B*_*i *_to obtain two new leaves *N' *and *N" *is defined as

$$infoGain(N,B_{i})=weightedEntropy(N)-\sum_{n\in \{N^{\prime },N^{\prime \prime }\}}\frac{totalWeight(n)}{TotalWeight(N)}\cdot weightedEntropy(n).$$

The attribute *B*_*i *_with the largest weighted information gain is chosen as label for *N *and corresponding children nodes *N' *and *N" *are added. The tree grows this way until no pair of node and attribute yields a positive information gain. This is a very loose stopping criterion and trees learned this way tend to be very large.

In order to avoid the problem of overfitting, a method called reduced-error pruning is used [21]. It uses a separate validation data set to prune the tree, and each split node in the tree is considered to be a candidate for pruning. When pruning a node, a operation called subtree replacement is performed, which involves removing the subtree rooted at that node and replacing the subtree with a single leaf. Whether pruning is performed depends on the classification accuracy obtained by the unpruned tree and by the pruned tree over the validation set. Pruning will cause the accuracy over the training data set to decrease; but it may increase the accuracy over the test data set.

## Results

Our approach was used to identify tissue-specific CRMs in human. First, we show, using a small set of experimentally verified tissue-specific CRMs, that our approach is able to discriminate between modules involved in different tissues. Then, we apply our method to a larger data set consisting of more than 6000 putative CRMs associated to genes specifically expressed in one of ten tissues, and show that interesting combinations of transcription factors can be linked to tissue-specific expression.

### Data sets

We used a set of cis-regulatory modules predicted in the human genome by Blanchette et al. [12], based on a set of 481 position weight matrices from Transfac 7.2 [22]. The modules are available from the PReMod database [11]. Criteria used for the PReMod predictions include inter-species conservation of binding site predictions and homotypic clustering of binding sites. The complete data set consists of more than 100,000 predicted CRMs, but only subsets of those were used (see below). For each predicted module *M*^*m*^, the predicted binding affinity $A_{f}^{m}$ is represented by the negative logarithm of the p-value of the binding site weighted density for factor Φ_*f *_in module *M*^*m*^, as reported in PReMod. Gene expression data came from the GNF Atlas 2 data set [14], downloaded from the UCSC Genome Browser [23]. A gene _*g*_*G *was identified as "expressed" (i.e. Egt = 1) if and only if its expression level was at least two standard deviations above its mean expression level, over the 79 tissues for which data was available.

Only 231 of the 481 Transfac PWMs were confidently linked to transcription factors for which GNF expression data is available. Only these |ℳ| = 231 PWMs were considered in our analysis. Some transcription factors are actually linked to several different PWMs, but our approach actually seems to take advantage of this to improve the quality of the predictions (see below).

### Validation experiments

We first use a set of experimentally verified tissue-specific CRMs, together with a set of negative control regions, to validate our algorithm. To further evaluate the performance of our approach, we compare our results with the results obtained with several simpler classifiers.

#### Validation data sets

To demonstrated the ability of our approach to identify tissue-specific regulatory modules, we used it to discriminate between known liver-specific CRMs, known erythroid-specific CRMs, and other modules not likely to be involved in these two cell types. Each validation data set was composed of five subsets:

1. knownLiver: 11 experimentally verified liver-specific modules [3].

2. knownErythroid: 22 experimentally verified erythroid-specific modules [24].

3. putativeLiver: A set of 31 PReMod modules located in the vicinity of the genes associated to the knownLiver modules. These modules are possibly involved in liver-specific regulation and are included only to help the Bayesian network learning the association between a module's binding site composition and tissue-specificity of the target gene.

4. putativeErythroid: A set of 46 PReMod modules similar to (3) but for erythroid.

5. negative: For each knownErythroid or knownLiver module with associated closest gene *g*, a set of *r*_*neg *_(see below) PReMod modules associated to genes that are expressed in neither erythroid nor liver is randomly selected and artificially associated to gene *g*. These are modules that, if placed in the vicinity of gene *g*, would be unlikely to cause liver or erythroid-specific expression.

The ratio *r*_*neg *_of the number of negative modules to the number of known modules determines in part the difficulty of the classification task. Two types of validation data sets were thus created: In our 1X experiment (see below), we used *r*_*neg *_= 1, whereas in our 2X data set, we used *r*_*neg *_= 2.

Each 1X data set thus contains 143 modules, each of which was considered as a possible liver or erythroid specific. The complete data set consists of 2 × 143 = 286 module-tissue pair, of which 11 + 22 = 33 are positive examples, 99 are negative examples (all the knownLiver modules when considered in the erythroid cell type, all the knownErythroid modules when considered in liver, and all the negative modules in both tissues). The 2X datasets are similar, except that they are noisier because they contain 165 negative examples.

#### Three simple classifiers

To assess the quality of our method, we compare it to three other simpler approaches. The first classifier, called the *expressionOnly *classifier, simply predicts that any module located next to a gene that is expressed in a given tissue is a tissue-specific module for that tissue. That is, the binding site content of the module is ignored, and only the expression _*g*_*E *is used to make the prediction.

The second simple classifier, called *SupervisedNaiveBayes*, is a classical supervised Naive Bayes approach that takes as input a simplified, observable version of the *B *variables, where we set $B_{m}^{f}$ = *F*_*m*_·*A*^*f*^, as well as the expression of the target gene Etg and is trained to distinguish between labeled positive and negative examples (see Appendix 4 for the complete details). Finally, the third simple classifier, called *NaiveBayesInNet*, is a version of our Bayesian Network classifier in which the regression tree representing the conditional probability of *R *is replaced by a Naive Bayes classifier, but where the rest of the structure is preserved. See Appendix 5 for more details.

#### Validation results

**Figure 3:**
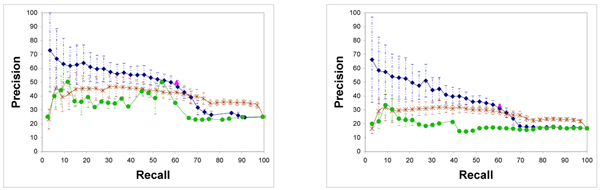
The precision v.s. recall curve for the 1*X *(left) and 2*X *(right) data sets, where precision = *TP*/(*TP *+ *FP*) and recall = *TP*/(*TP *+ *FN*). The blue curve (diamond markers) is generated from the results of our approach, the brown curve (× markers) is generated from the results of the *Supervised-NaiveBayes *approach (see Appendix 4), and the green curve (circle markers) is generated from the results of the *NaiveBayesInNet *classifier (see Appendix 5). The pink triangle shows the result obtained by the expressionOnly classifier. Error bars denote one standard deviation of the precision, over 100 random choices of negative examples. The increase in the standard deviation on precision at lower recall is due to the small number of predictions made for these thresholds.

One hundred different runs of our EM-like algorithm were done on 1X and 2X datasets, each time with a different sample of negative modules. Each run used 100 EM-like iterations (taking approximately 10 minutes of running time), which was sufficient to achieve convergence, although different runs converge to slightly different likelihoods and regression trees (see Additional File 1). Since we do not know which of the putativeLiver and putativeErythroid CRMs are actually tissue-specific modules, we evaluate the performance of our algorithm based only on the positive and the negative modules. For each run, the network with the best likelihood over 100 EM-like iterations is used to compute Pr[^*t*^*R*^*m*^|**A**, **E**, **F**] for all examples and a module-tissue pair is predicted positive if this probability exceed some threshold *t*. The resulting precision-recall curve, averaged over all 100 runs, is shown in Figure 3, for both the 1X and 2X data set.

Since 13 out of the 33 known CRMs have target genes expressed neither in liver nor in erythroid (based on our discretization of expression data), the *ExpressionOnly *classifier yields a recall = 60.6% and precision = 50% on the 1X data set, but only precision = 33% on the 2X data set.

As seen from the curves, our method significantly outperforms both the Naive Bayes-based approaches for mid- to high-precision predictions. Our method can improve the precision to 72% for the 1*X *data sets and 66.2% for the 2*X *data sets. Notice that the highest precision for the 2*X *data sets remains close to that for the 1*X *data sets, although almost twice as many negative examples are considered. This indicates that our approach provides a way to improve the precision of prediction by combining the sequence data and the expression data.

#### Regression trees

**Figure 4:**
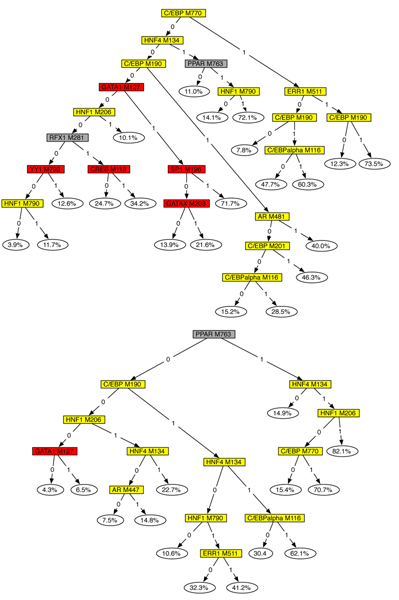
The regression tree generated by the iteration with the best likelihood for a 1*X *(top) and 2X (bottom) data sets. Internal nodes corresponding to liver-specific transcription factors are colored yellow, and those corresponding to erythroid-specific factors are red.

Figure 4 shows the regression trees generated from one run for the 1*X *and 2*X *data sets. Each internal node tests the value of an attribute *B*_*f*_, which indicates whether factor Φ_*f *_is predicted to bind the module in the tissue under consideration. Each leaf shows the conditional probability predicted, which is the probability of *R *= 1 on the condition specified by the path from to root to the leaf.

The tree structure indicates what are the most important TFs or combinations of TFs for explaining liver-specific and erythroid-specific expression. Our algorithm successfully detects most known liver-specific TFs and combinations of thereof, like *HNF1 *+ *HNF4*, *HNF1 *+ *C/EBP*, and *HNF4 *+ *C/EBP*, which are reported in the literature [3]. The erythroid-specific TF *GATA1 *is also reported in the trees. The trees do not contain many erythroid-specific nodes, firstly because there are only two TFs (GATA1 and NF-E2) that are erythroid-specific based on our expression data, and secondly because NF-E2 has very few predicted binding sites on the genome. We observe from the trees that the leaves associated with TF combinations usually have higher regulatory probabilities than the leaves associated with individual TFs. This indicates that the ability to identify TF combinations is key to being able to identify cis-regulatory modules. We emphasize that the trees were obtained without any prior information about which of the 231 PWMs used are involved in liver or erythroid-specific expression.

Notice that TF *PPAR *is reported in our trees. *PPAR *is indeed an important factor regulating expression in liver [25], but was absent from Krivan and Wasserman's paper [3] from which we obtain the known liver-specific CRMs. Most importantly, the expression of *PPAR *is low in both liver and erythroid, so ^*erythroid*^*F*_*PPAR *_= ^*liver*^*F*_*PPAR *_= 0. This shows that our approach is robust to noise in the expression data of TFs, provided the association between the binding sites in modules and the target gene's expression is sufficiently high. Finally, we note the unexpected selection of several different matrices for the same transcription factor along the same path in the tree (for example C/EBP M770 and M190 on the tree obtained for the 1X data set on Figure 3). This is caused by the fact that these matrices are quite actually different from each other, but the presence of sites for both matrices increases the association to the target gene's expression.

## Genome-wide CRM prediction in ten tissues

We next extended our analysis to ten different tissues from the GNF Atlas2: T = {brain, erythroid, thyroid, pancreatic islets, heart, skeletal muscle, uterus, lung, kidney, and liver}. 923 genes are specifically expressed (i.e. Egt = 1) in at least one of these tissues and a total of 6278 modules are associated to these genes. We thus trained our Bayesian network on a set of 10 × 6, 278 = 62, 780 (module, tissue) pairs. Ten parallel runs of 100 EM-like iterations were performed from different random initializations, each taking approximately 24 hours.

**Figure 5:**
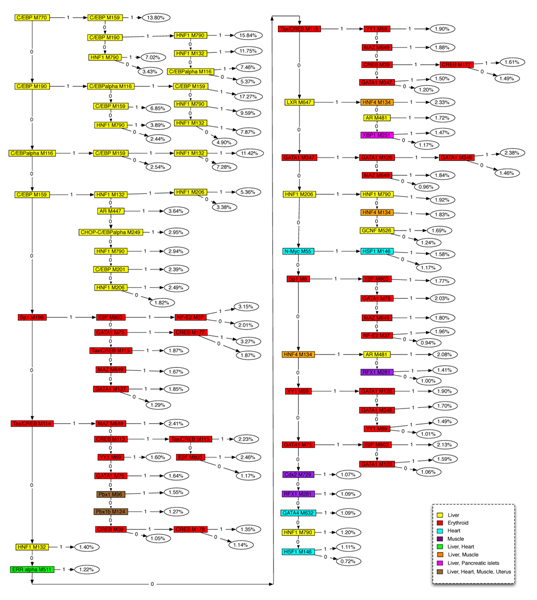
Regression tree obtained from the best of ten runs on the set of 6,278 modules and 10 tissues. Nodes are colored based on the tissue in which a particular factor is expressed.

The regression tree obtained obtained from the best run is shown in Figure 5. We can clearly observe from the tree that the positive assignments along each path leading to a leaf typically consists of TFs expressed in the same tissue. Several known tissue-specific combinations of TFs are recovered in the tree, such as *C/EBP *+ *HNF1 *and *C/EBP *+ *HNF4 *in liver. Also, many new and potentially meaningful TF combinations are predicted, such as *C/EBP *+ *AR *in liver and *Tax/CREB *+ *GATA1 *in erythroid.

The tree only contains the TFs expressed in four tissues: liver, erythroid, heart, and skeletal muscle. The other six tissues are not represented in the tree because of one of the following reasons: (1) The TFs that regulate the genes expressed in those tissues have low expression levels. (2) These TFs do not have strict requirements for sequence affinity, so the binding scores of their matrices are low. It is also possible that there are no PWMs for such TFs. (3) The expression of genes in those tissues are controlled by post-transcriptional regulation instead of tissue-specific TFs.

The complete set of tissue-specificity predictions are available at http://www.mcb.mcgill.ca/~xiaoyu/tissue-specificModule.

### Statistical analysis of TF combinations

The regression trees obtained in the 10 runs vary substantially in their structure but share many of their factors and combination of factors. The frequency with which factors or combination of factors are found in these trees is an indication of their role in regulating tissue-specific expression. A pair of factors is said to co-occur in a regression tree if the tree contains a path along which both factors take value 1. As seen in Tables 1 and 2, several factors and pairs of factors are consistently identified as part of the tree. Most TFs found are either known to be directly involved in tissue-specific regulation (in bold in Table 1, or to be essential for the expression of certain genes in the given tissues, but to also have other non-tissues-specific roles (normal font in Table 1).

**Table 1:** Significant TFs selected in the 10-tissue experiment.

Transcription factor	Number of occurrences	Expressed in tissue(s)	Support from literature
HNF1	10	Liver	**[27]**
C/EBP	10	Liver	**[27]**
C/EBPalpha	10	Liver	**[27]**
AR	8	Liver	**[28]**
Sp1	8	Erythroid	[29]
E2F	7	Erythroid	[30]
MAZ	7	Erythroid	[31]
Tax/CREB	7	Erythroid	[32]
GATA1	7	Erythroid	**[33]**
ERRalpha	6	Liver, Heart	**[34]**
CREB	6	Erythroid	[32]
HNF4	6	Liver, Skeletal muscle	**[27]**
YY1	5	Erythroid	[35]
Cdx2	5	Skeletal muscle	
LXR	5	Liver	**[36]**
GATA4	5	Heart	**[37]**
RFX1	5	Skeletal muscle	
XBP1	5	Liver, Pancreatic islets	**[38]**
N-Myc	5	Heart	[39]

Transcription factors present in the regression tree in at least five of the 10 runs. References in bold refer to papers arguing for tissue-specificity of the given factor in the given tissue, whereas those in normal font point to papers showing the involvement of the given TF for the proper expression of some gene(s) expressed in the given tissue, but where the TF is not tissue-specific.

**Table 2:** Significant TFs pairs selected in the 10-tissue experiment.

Transcription factor pair	Number of occurrences	Expressed in tissue(s)
C/EBP + C/EBPalpha	10	Liver
HNF1 + C/EBP	7	Liver
HNF1 + C/EBPalpha	5	Liver
Tax/CREB + MAZ	5	Erythroid
Sp1 + E2F	4	Erythroid
C/EBP + AR	4	Liver
C/EBP + CHOP-C/EBPalpha	4	Liver
CREB + Tax/CREB	4	Erythroid
GATA1 + Sp1	4	Erythroid
GATA1 + CREB	4	Erythroid
GATA1 + YY1	4	Erythroid
AR + LXR	3	Liver
CREB + MAZ	3	Erythroid
HNF4 + AR	3	Liver
Tax/CREB + E2F	3	Erythroid
GATA1 + E2F	3	Erythroid
GATA1 + Tax/CREB	3	Erythroid
YY1 + Tax/CREB	3	Erythroid
YY1 + CREB	3	Erythroid
Sp1 + Tax/CREB	3	Erythroid

Transcription factor pairs present together on the same path of the regression tree in at least three of the 10 runs.

### Predicting gene tissue-specificity

To further validate our module tissue-specificity predictions, we investigated whether a gene's tissue-specific fine-grain expression level could be predicted based on the modules regulating it. To this end, for each tissue t, we separated genes between highly expressed Pgt) and low expressed (Egt = 0). Let Pgt be the maximum of the predicted regulatory activity ^*t*^*R*^*m *^of the modules associated to gene *g*. We asked whether Pgt is predictive of the raw, non-thresholded expression level of gene *g*. In the case of genes with Egt = 0, such a correlation would show that we are able to detect tissue-specific genes even if their expression level is below the threshold. For genes with Egt = 1, this correlation would show that genes with very high tissue-specific expression levels are associated to stronger module predictions than those that barely meet our threshold. We note that in both cases, such a correlation could not be explained by any kind of training artifact, since raw expression data is not part of the input.

Considering genes showing tissue-specific expression (Egt = 1), we find that eight of the ten tissues considered (all but "whole brain" and "erythroid") exhibit a positive correlation between Pgt and the raw gene expression. Somewhat surprinsingly, the correlation is strongest for thyroid (p-value = 0.028) and skeletal muscle (p-value = 0.015), two factors that were relatively poorly represented in our regression tree. Among genes with Etg = 0, the correlation is weaker but is positive in seven of the ten tissues (all except heart, skeletal muscle, and liver). These results indicate that our predictions yield a weak predictor of gene tissue-specificity. Clearly, it is easier to predict modules responsible for a gene's observed tissue-specificity than to predict the tissue-specificity of a gene based on its modules.

## Discussion and conclusion

The approach we introduced here is the first to integrate binding site predictions and tissue-specificity of expression of both transcription factors and target genes to predict cis-regulatory modules involved in regulating tissue-specific gene expression. By introducing a regression tree at the heart of the network and deriving practical algorithms to train it, we are able to accurately identify important combinations of transcription factors regulating gene expression in a tissue-specific manner. The algorithms derived for learning this type of network will undoubtedly be applicable to a wide range of problems.

Many of the choices made in the design of the Bayesian network were made for computational practicality reasons. As we improve the learning algorithm, it will become possible to use real-numbered expression measurements.

Furthermore, our network could easily be extended by introducing additional sources of information as observed variables. For example, ChIP-chip and other binding assay data, when available, can be used to affect our belief in $\begin{array}{c} t \end{array}B_{f}^{m}$. Reporter assays and DNA accessibility assays could be used to modify our belief in ^*t*^*R*^*m*^. If modeled correctly, these types of experimental data may greatly increase the accuracy of our predictions, not only for the modules or the factors for which data is available, but also for other regions or factors associated to similar functions.

The approach we described is potentially applicable to a wide range of data sets. While the relative inefficiency of the current learning algorithm prevented us from analyzing the complete set of tissue-specific expression from GNF, it is clear that this analysis, involving 79 tissues, would yield a wealth of information. Another possible application is to identify and characterize cis-regulatory modules involved in time and tissue-specific regulation during fish development. The large body of in situ hybridization data available in zebrafish [26] would provide an excellent basis for this analysis.

## Competing interests 

The authors declare that they have no competing interests.

## Authors' contributions

XC designed and implemented the prediction algorithms, obtained all the results presented, and participated to the manuscript redaction. MB contributed to the original idea, the mathematical formulation and the redaction. All authors read and approved the final manuscript.

## Appendix 1. Calculation of Pr[*B*_*f*_|*A*_*f*_, *F*_*f*_]

Pr[*B*_*f*_|*A*_*f*_, *F*_*f*_] is the probability of TF Φ_*f *_binding a genomic region, given its observed expression *F*_*f *_and its binding affinity *A*_*f *_for the region. Modeling this relationship is challenging because it is unclear how *B*_*f*_, a binary variable, should depend on *A*_*f*_, a continuous variable, in the presence of the observed expression *F*_*f*_. For this reason, we derive this probability from a set of other probabilities distributions that are easier to model, specifically Pr[*A*_*f*_|*B*_*f *_= 1], the affinity score distribution of sites that are bound.

Recall that ${\hat{F}}_{f}$ is defined as the actual expression of factor Φ_*f*_. Note first that

$$\begin{array}{ccc} \mathrm{Pr}[B_{f}|F_{f},A_{f}] & = & \sum_{e^{\prime }\in \{0,1\}}\mathrm{Pr}[B_{f}|F_{f},A_{f},{\hat{F}}_{f}=e^{\prime }]\cdot \mathrm{Pr}[{\hat{F}}_{f}=e^{\prime }|F_{f},A_{f}]\\ & = & \sum_{e^{\prime }\in \{0,1\}}\mathrm{Pr}[B_{f}|A_{f},{\hat{F}}_{f}=e^{\prime }]\cdot \mathrm{Pr}[{\hat{F}}_{f}=e^{\prime }|F_{f}]\\ & = & \sum_{e^{\prime }\in \{0,1\}}\mathrm{Pr}[B_{f}|A_{f},{\hat{F}}_{f}=e^{\prime }]\cdot \mathrm{Pr}[F_{f}|{\hat{F}}_{f}=e^{\prime }]\cdot \mathrm{Pr}[{\hat{F}}_{f}=e^{\prime }]/Z\\ & = & \sum_{e^{\prime }\in \{0,1\}}\mathrm{Pr}|A_{f}|B_{f},{\hat{F}}_{f}=e^{\prime }]\cdot \mathrm{Pr}[B_{f}|{\hat{F}}_{f}=e^{\prime }]\cdot \mathrm{Pr}[F_{f}|{\hat{F}}_{f}=e^{\prime }]\cdot \mathrm{Pr}[{\hat{F}}_{f}=e^{\prime }]/Z^{\prime } \end{array}$$

for some appropriately chosen constants *Z *and *Z'*. The distribution of Pr[*F*_*f*_|${\hat{F}}_{f}$] is described in Section 'Conditional distributions of *E *and *F'*, and the prior probability Pr[${\hat{F}}_{f}$] is approximated by the prior probability of the observed variable Pr[*F*_*f *_]. So all that remains is to define Pr[*A*_*f*_|*B*_*f*_, ${\hat{F}}_{f}$] and Pr[*B*_*f*_|${\hat{F}}_{f}$].

Because a TF can bind only if it is expressed, we have

$$\text{Pr}[A_{f}|B_{f}=1,{\hat{F}}_{f}=0]$$

$$=\text{Pr}[A_{f}|B_{f}=1,{\hat{F}}_{f}=1]=\text{Pr}[A_{f}|B_{f}=1]$$

When ${\hat{F}}_{f}=0$, the event *B*_*f *_= 0 yields no information on *A*_*f*_, so

$$\text{Pr}[A_{f}|B_{f}=0,{\hat{F}}_{f}=0]=\text{Pr}[A_{f}]$$

where the prior probability Pr[*A*_*f*_] is estimated from the data using a histogram approach.

Notice that

$$\text{Pr}[A_{f}]=\text{Pr}[A_{f},B_{f}=0,{\hat{F}}_{f}=0]+\text{Pr}[A_{f},B_{f}=0,$$

$${\hat{F}}_{f}=1]+\text{Pr}[A_{f},B_{f}=0,$$

$${\hat{F}}_{f}=0]+\text{Pr}[A_{f},B_{f}=1,$$

$${\hat{F}}_{f}=1]$$

We thus obtain

$$\mathrm{Pr}[A_{f}|B_{f}=0,{\hat{F}}_{f}=1]=\frac{A-B}{\mathrm{Pr}[B_{f}=0,{\hat{F}}_{f}=1]}$$

where

$$A\text{Pr}[A_{f}]\text{·}(1-\text{Pr}[B_{f}=0,{\hat{F}}_{f}=0])$$

$$B=\text{Pr}[A_{f}|B_{f}=1\text{·}(\text{Pr}[B_{f}=1,{\hat{F}}_{f}=0]+\text{Pr}[B_{f}=1,$$

$${\hat{F}}_{f}=1])$$

and where

$$\text{Pr}[B_{f}=x,{\hat{F}}_{f}=y]=$$

$$\text{Pr}[B_{f}=x|{\hat{F}}_{f}=y]\cdot$$

$$\text{Pr}[{\hat{F}}_{f}y]$$

We assume that Pr[*A*_*f*_|*B*_*f *_= 1] follows a normal distribution with parameters *μ*_*f *_and $\sigma_{f}^{2}$ that are optimized during the EM-like algorithm (see Appendix 3). Pr[*F*_*f*_|${\hat{F}}_{f}$] and Pr[${\hat{F}}_{f}$] have all been previously defined.

Finally, Pr[*B*_*f*_|${\hat{F}}_{f}$] is represented by a fixed CPT:

$$\mathrm{Pr}[B_{f}|{\hat{F}}_{f}]=\begin{array}{ccc} & B_{f}=0 & B_{f}=1\\ {\hat{F}}_{f}=0 & 1 & 0\\ {\hat{F}}_{f}=1 & 1-\gamma & \gamma \end{array}$$

where *γ *= 0.01 is a parameter that indicates the prior probability that an expressed TF will bind a generic genomic region.

## Appendix 2. Formulas for E-step

### Calculation of Pr[*R*^*m*^|**A**, **E**, **F**]

Let *X *be the set of modules associated with the same gene *g*. Let *S *= ∑_*r*∈*X*_*R*^*r*^, we where

Pr⁡[Rm|A,E,F]=1/Z⋅∑b,e,sPr⁡[Rm,S=s,B=b,E^g=e,A,E,F]=1/Z⋅(∑bPr⁡[Rm|Bm=b]∏fPr⁡[Bfm=bf|Afm,Ff]).=(∑ePr⁡[Eg|E^g=e]∑sPr⁡[E^g=e|S=s]⋅Pr⁡[S=s|Rm,AX−m,F])

where

• The regression tree allows an efficient computation of the first sum:

$$\sum_{b}\text{Pr}[R^{m}|B^{m}=b]\prod_{f}\text{Pr}[B_{f}^{m}$$

$$=b_{f}|A_{f}^{m},F_{f}]$$

$$=\sum_{\text{leaf}l}P(R|l)\cdot \prod_{\text{assignments}\Lambda \text{in}\pi (l}\text{Pr}[\Lambda |A_{f}^{m},F_{f}]$$

• Pr[$B_{f}^{m}$|$A_{f}^{m}$, *F*_*f*_] has been defined in Appendix 1;

• Pr[_*g*_*E*|_*g*_$\hat{E}$] is represented by a CPT described in Section 'Conditional distributions of *E *and *F'*;

• Pr[_*g*_$\hat{E}$|*S *= *s*] is defined by the sigmoid function 1/(1 + *e*^-*b*(*s*-*a*)^).

Further noting that

Pr[*S *= *s*|*R*^*m*^, **A**^*X*-*m*^, **F**] = Pr[∑_*r*∈*X*-*m*_*R*^*r *^= *s *- *R*^*m*^|**A**^*X*-*m*^, **F**], we can calculate Pr[*S *= *s*|*R*^*m*^, **A**^*X*-*m*^, **F**] by using a simple dynamic programming.

### Calculation of Pr[$B_{f}^{m}$|**A**, **B**, **F**]

$$\begin{array}{l} \mathrm{Pr}[B_{f}^{m}|A,E,F]=\sum_{r}Pr[B_{f}^{m}|A^{m},E,F,R^{m}=r]\cdot \mathrm{Pr}[R^{m}=r|A,E,F]\\ \mathrm{Pr}[B_{f}^{m}|A^{m},E,F,R^{m}]=\frac{Pr[B_{f}^{m}|A_{f}^{m},F_{f}]\cdot \mathrm{Pr}[R^{m}|B_{f}^{m}]}{Z} \end{array}$$

Note that Pr[$B_{f}^{m}$|$A_{f}^{m}$, *F*_*f*_] has been defined in Appendix 1. Furthermore, we can estimate Pr[*R*^*m*^|$B_{f}^{m}$] from the data

$$\mathrm{Pr}[R^{m}|B_{f}^{m}]=\frac{\sum_{m,t}\mathrm{Pr}[\begin{array}{c} t \end{array}R^{m}|A,E,F]\cdot \mathrm{Pr}[\begin{array}{c} t \end{array}B_{f}^{m}|A,E,F]}{\sum_{m,t}\mathrm{Pr}[\begin{array}{c} t \end{array}B_{f}^{m}|A,E,F]}$$

where Pr[$\begin{array}{c} t \end{array}B_{f}^{m}$|**A**, **E**, **F**] takes the values calculated from the previous iteration.

We thus get

$$\begin{array}{ccc} \mathrm{Pr}[B_{f}^{m}|A,E,F] & = & 1/Z\cdot \sum_{r}\mathrm{Pr}[A_{f}^{m}|B_{m}^{f},F_{f}]\cdot \mathrm{Pr}[B_{f}^{m}|F_{f}]\cdot \\ & & \mathrm{Pr}[R^{m}=r|B_{f}^{m}]\cdot \mathrm{Pr}[R^{m}=r|A,E,F] \end{array}$$

where Pr[$A_{f}^{m}$|$B_{f}^{m}$, *F*_*f*_] is obtained as in Appendix 1 and

$$\text{Pr}[B_{f}^{m}|F_{f}]$$

$$=\sum_{\mathrm{e'}\in \{0,1\}}\text{Pr}[B_{f}^{m}|F_{f},$$

$${\hat{F}}_{f}=\mathrm{e'}]\cdot$$

$$\text{Pr}[{\hat{F}}_{f}=\mathrm{e'}|F_{f}]/Z.$$

### Calculation of Pr[$\hat{E}$|**A**, **F**, **E**] and Pr[$\hat{F}$|**A**, **F**, **E**]

Although $\hat{E}$ is a hidden variable, its posterior probability distribution does not need to be estimated, because we sum over all its possible values when computing Pr[*R*^*m*^|**A**, **F**, _*g*_*E*]. The same holds for $\hat{F}$ in Pr[*B*_*f*_|*A*_*f*_, *F*_*f*_].

## Appendix 3. Parameter re-estimation (M-step)

Pr[*A*_*f*_|*B*_*f *_= 1] is assumed to follow a normal distribution *N*(*μ*_*f*_, $\sigma_{f}^{2}$).

Parameters *μ*_*f *_and *σ*_*f *_are re-estimated as follows:

(1) $$\begin{array}{lll} \mu_{f} & \leftarrow & \frac{\sum_{m,t}\mathrm{Pr}[\begin{array}{c} t \end{array}B_{f}^{m}=1|A,E,F]\cdot A_{f}^{m}}{\sum_{m,t}\mathrm{Pr}[\begin{array}{c} t \end{array}B_{f}^{m}=1|A,E,F]}\\ \sigma_{f}^{2} & \leftarrow & \frac{\sum_{m,t}\mathrm{Pr}[\begin{array}{c} t \end{array}B_{f}^{m}=1|A,E,F]\cdot {\left(A_{f}^{m}\right)}^{2}}{\sum_{m,t}\mathrm{Pr}[\begin{array}{c} t \end{array}B_{f}^{m}=1|A,E,F]}-\mu_{f}^{2} \end{array}$$

In order to avoid overstepping the local maximum, we use small steps when updating the values of *μ*_*f *_and *σ*_*f*_. Instead of replacing the old values with the new values calculated from Equation 1, we use a hybrid of the old values and the new values, weighted according to the step size.

$$\begin{array}{lll} \mu_{f} & \leftarrow & (1-\alpha )\cdot \mu_{f}^{old}+\alpha \cdot \mu_{f}^{new}\\ \sigma_{f}^{2} & \leftarrow & (1-\alpha )\cdot \sigma_{f}^{2,old}+\alpha \cdot \sigma_{f}^{2,new} \end{array}$$

where *α *= 0.1 is the step size.

The following parameters have values that remain fixed throughout the execution of the EM-like algorithm. Their value has been chosen empirically to optimize the quality of the results.

1. Parameters for Pr[*E*|$\hat{E}$] and Pr[*F*|$\hat{F}$]: *α*_*E *_= *β*_*E *_= *α*_*F *_= *β*_*F *_= 0.1

2. Parameters for Pr⁡[E^g|Rr1,Rr2,...]:

*a *= 0.8, *b *= 10 in validation experiments (small data sets), and

*a *= 0.4, *b *= 10 in discovery experiments (large data set).

3. Parameters for Pr[*B*_*f*_|${\hat{F}}_{f}$]: *γ *= 0.01.

## Appendix 4. The *SupervisedNaiveBayes *classifier

A naive Bayes classifier was trained to discriminate between positive and negative (module, tissue) pairs. First, the affinity $A_{i}^{m}$ is discretized as 1 if and only if its value is at least one standard deviation above the mean of *A*_*i*_, over all 100,000 putative modules from PReMod. The Naive Bayes network takes as input the following set of attributes: $F_{1}\cdot A_{1}^{m},...,F_{\left|F\right|}\cdot A_{\left|F\right|}^{m}$, and *E*_*g*_. The precision-recall curves from Figure 3 were the result of a 11-fold cross-validation experiment.

## Appendix 5. The *NaiveBayesInNet *classifier

The *NaiveBayesInNet *classifier is a Bayesian Network identical to the main classifier presented in this paper, except that a Naive-Bayes-like approach replaces the probability tree representing Pr[*R*|*B*_1_,...,*B*_Φ_]. More specifically, it assumes Pr[*R*|*B*_1_,...,*B*_Φ_] = ∏_*f*=1...Φ_Pr[*B*_*f*_|*R*]/*Z*.

At each iteration of the EM-like algorithm, Pr[*B*_*f*_|*R*] is estimated as $\mathrm{Pr}[B_{f}=a|R=b]=\frac{\sum_{t=1}^{\left|T\right|}\sum_{m=1}^{\left|ℳ\right|}\mathrm{Pr}[\begin{array}{c} t \end{array}B_{f}^{m}=a|A,E,F]\cdot \mathrm{Pr}[\begin{array}{c} t \end{array}R_{f}^{m}=b|A,E,F]}{\sum_{t=1}^{\left|T\right|}\sum_{m=1}^{\left|ℳ\right|}\mathrm{Pr}[\begin{array}{c} t \end{array}R_{f}^{m}=b|A,E,F]}$. Then, estimating Pr[*R*|**A**, **F**, **E**] requires a summation over all $2^{\left|ℱ\right|}$ possible values of the **B **variables (the simplification afforded by the regression tree cannot be applied here). To make the computation practical, we instead fix the value of the **B **to their maximum likelihood estimates and use these fixed values to estimate Pr[*R*|**A**, **F**, **E**]. The approach was trained and evaluated using exactly the same methodology as for the Bayes network approach using regression trees.

## Supplementary Material

Additional file 1 The logarithms of the likelihoods for the 2*X *validation experiments in three different randomly selected runs. Different colors represent different runs. Click here for file
