# Supplementary figures and images for: Prediction of tissue-specific cis-regulatory modules using Bayesian networks and regression trees
Source: BMC Bioinformatics. 2007 Dec 21;8(Suppl 10):S2. doi: 10.1186/1471-2105-8-S10-S2 (PMC2230503; doi:10.1186/1471-2105-8-S10-S2)

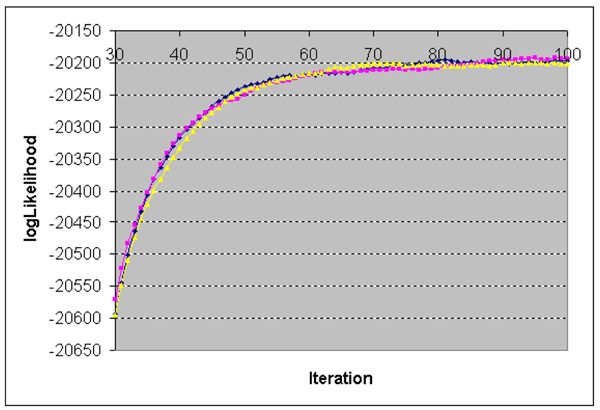

Supplement: Additional file 1 — The logarithms of the likelihoods for the 2X validation experiments in three different randomly selected runs. Different colors represent different runs. [file 1471-2105-8-S10-S2-S1.jpg]
